# Supplementary material for: Acute Care of Older Patients with COVID-19: Clinical Characteristics and Outcomes
Source: Geriatrics (Basel). 2020 Sep 27;5(4):65. doi: 10.3390/geriatrics5040065 (PMC7709587; doi:10.3390/geriatrics5040065)
Supplement: Supplementary file 1 [file geriatrics-05-00065-s001.pdf]

Supplementary material :

Figure S1: Multivariate Cox regression for prognostic factors.

Multivariate Cox regression analysis of risk factors associated with acute respiratory failure. Significant risk factors in the univariate cox regression analysis were added in the model.

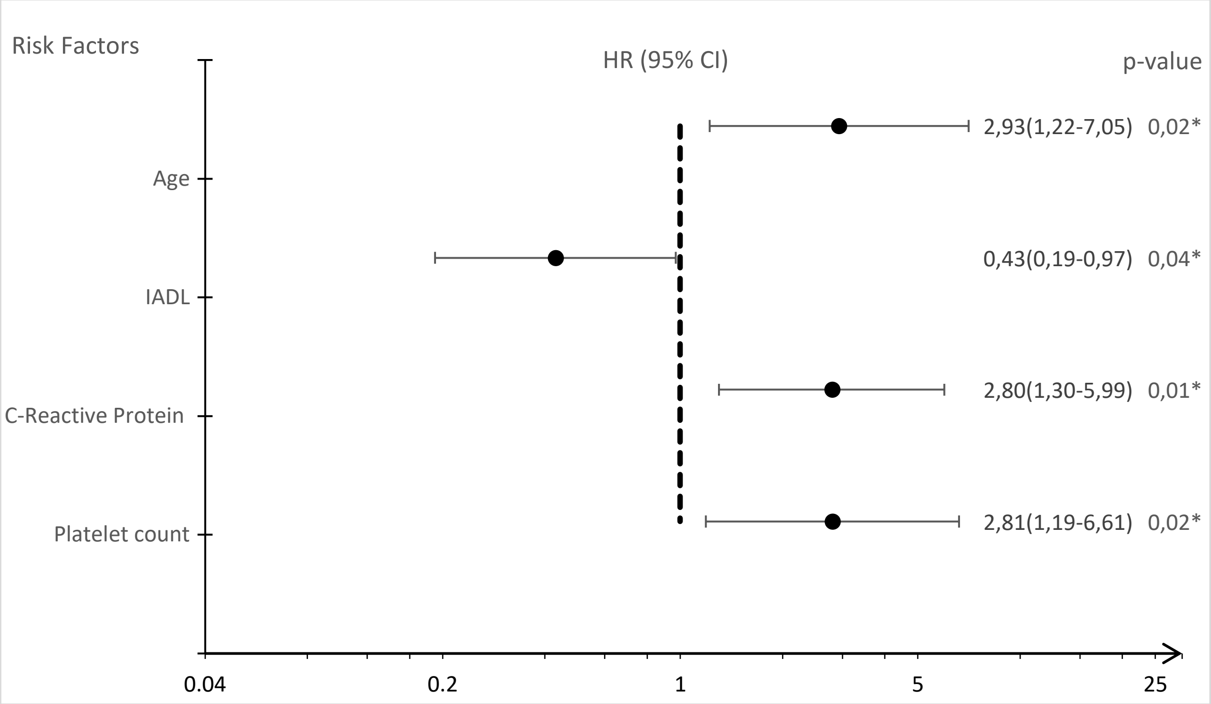

Abbreviations: IADL, Instrumental activities of daily living; CRP: C-Reactive protein level; HR, hazard ratio. CI: Confidence interval.

*$P < 0.05$

Table S1. Baseline characteristics of hospitalized older patients with COVID-19 stratified by age

	Survivors (n=77)			Non-survivors (n=17)		
	Age group					
Characteristics (n=94)	<85 years	≥85 years	p-value	<85 years	≥85 years	p-value
**Socio-demographic**						
Gender, Female	18(48.6)	23(57.5)	0.44	4(100)	7(63.64)	0.09
**Marital status** (n=93)			0.06			0.03*
Single	5(13.9)	1 (2.5)		2(50)	0	
Married	17 (47.2)	18 (45)		2(50)	5(38.5)	
Widowed	9 (25)	19 (47.5)		0	7(53.9)	
Divorced	5 (13.9)	2 (5)		0	1(7.7)	
**Living Location**			0.55			0.54
Private house-hold	26 (70.3)	27 (67.5)		2(50)	9(69.2)	
Assisted living	4 (10.8)	2 (5)		0	1(7.7)	
Nursing home	7 (18.9)	10 (25)		2(50)	32(23.1)	
Other	0	1 (2.5)		0	0	
**Home health care services**						
Nurse	20 (54)	30 (75)	0.05*	2(50)	6(46.1)	0.89
Home health aides	22 (59.5)	29 (72.5)	0.2	2(50)	(38.5)	0.68
**Health conditions**						
**Cumulative Illness Rating Scale (CIRS-G)**						
Total score	11.3±4.5	13±5.3	0.14	15±4.55	12.4±5.02	0.37
Cardiac Disease	13 (35.1)	22 (55)	0.08	1(25)	10(76.9)	0.06
Hypertension	24 (64.9)	28 (70)	0.63	3 (75)	10 (76.9)	0.94
Respiratory Disease	16 (43.2)	14 (35)	0.46	2(50)	3(23.1)	0.30
Diabetes Mellitus	6(16.2)	3 (7.5)	0.23	1(25)	1(7.69)	0.35
Dementia	18 (48.6)	16 (40)	0.44	3(75)	6(46.1)	0.31
**Medication**						
Number of medications	5.9± 2.8	6±3.1	0.81	8.2±5.9	7.9±3.9	0.9
Presence of ACEI or ARA	17 (45.9)	7 (17.5)	<0.01*	1(25)	8(61.5)	0.2
Presence of antiplatelet / anticoagulant therapy	14 (37.8)	24 (60)	0.05*	0	11(84.6)	<0.01*
**Functional assessment**						
**FIND**						0.34
Dependant	24(64.9)	36 (90)	0.03*	0	3(23.1)	
Frail	6 (16.2)	2 (5)		0	2 (15.4)	
Robust	7(18.9)	2(5)		4(100)	8(61.54)	
**ADL score(/6) ,** ≥1 Dependency	20 (54)	30 (75)	0.05	4(100)	7 (53.8)	0.09
**IADL score(/8),** ≥1 Dependency	21 (60)	37 (92.5)	0.01*	4(100)	8(61.5)	0.14
**Nutritional assessment**						
**Mini MNA**			0.04*			0.49
Normal nutritional status (12-14)	11(29.7)	3 (7.5)		0	3(23.1)	
Risk of malnutrition (8-11)	13 (35.1)	17 (42.5)		1(25)	4(30.8)	
Malnutrition (≤7)	13 (35.1)	20 (50)		3(75)	6(46.1)	
**Norton scale** (n=91)						0.55
<14 (Pressure ulcer risk)	12 (32.4)	14 (36.8)	0.68	2 (50)	4(33.3)	

Abbreviations: SD: Standard deviation; IQR : Inter quartile; ACEI= Angiotensin-converting enzyme inhibitor , ARA= angiotensin II receptor antagonists, CIRS-G: Cumulative Illness Rating Scale Geriatric; FiND: Frail Non-Disabled questionnaire; ADL : Activities of Daily Living; IADL : Instrumental Activities of Daily Living; Mini MNA: Mini Nutritional Assessment;BMI: Body Mass Index; *P<0.05.

Table S2. Multivariate analysis of risk factors associated with mortality in older patients with COVID-19 adjusted for gender.

Multivariate analysis		
Variable	OR (95%CI)	p-value
Gender, Female	1.75(0.51-5.9)	0.37
Onset of acute respiratory failure	4.6(1.2-17.7)	0.03*
Lymphopenia (<0.8G/L)	3.5(1.1-11.4)	0.03*

Abbreviations: OR: Odd ratios per standard deviation with 95% confidence interval
*Variables with P<0.05 in the multivariate analysis
